# New Active Biopolymers and Chitosan-Based Films from Non-Native Crayfish Shell

**DOI:** 10.3390/molecules31162819

**Published:** 2026-08-13

**Authors:** Rosa Zullo, Rosaria Lauceri, Alberto Zullo, Luigi Sorrentino, Angela Boggero, Lyudmila Kamburska, Andrea Lami, Silvia Zaupa, Maria Oliviero

**Affiliations:** 1 National Research Council-Water Research Institute (CNR-IRSA), Corso Tonolli 50, 28922 Verbania Pallanza, Italy; rosaria.lauceri@cnr.it (R.L.); angela.boggero@cnr.it (A.B.); lyudmilatodorova.kamburska@cnr.it (L.K.); andrea.lami@cnr.it (A.L.); silvia.zaupa@cnr.it (S.Z.); 2 Department of Sciences and Technologies, University of Sannio, Via De Sanctis, 82100 Benevento, Italy; albzullo@unisannio.it; 3 National Research Council-Institute for Polymers, Composites and Biomaterials (CNR-IPCB), Piazzale E. Fermi 1, 80078 Portici (Napoli), Italy; luigi.sorrentino@cnr.it (L.S.); maria.oliviero@cnr.it (M.O.); 4 National Biodiversity Future Center (NBFC), Piazza Marina 61, 90133 Palermo, Italy

**Keywords:** chitosan film, invasive species, mechanical properties, physicochemical characterization, antioxidant activity, astaxanthin, HPLC

## Abstract

Freshwater crayfish impact ecosystems and the food industry. The aim of the study was to valorize shells from the invasive crayfish, *Faxonius limosus*, to extract novel bioactive biopolymers and prepare biodegradable films, thereby contributing to economic and environmental sustainability. Chitin from crayfish shells was isolated and converted into chitosan through a new protocol to retain astaxanthin, with a shorter procedure, reduced use of organic solvent and energy consumption. The resulting biopolymers were used to prepare chitosan-based films. Thermal, spectroscopic, barrier properties, and surface microstructure of the biomaterials were identified. Spectrophotometric and chromatographic techniques were used on crayfish shells and biopolymers to track astaxanthin throughout the extraction and formulation processes. Crayfish chitosan film exhibited higher stiffness, enhanced thermal stability and water vapor barrier properties with respect to commercial chitosan films. 2,2′-azino-bis(3-ethylbenzothiazoline-6-sulfonic acid) and 2,2-diphenyl-1-picrylhydrazyl antioxidant assays showed that all crayfish biomaterials exhibited antioxidant activity. Astaxanthin greatly enhanced the antioxidant properties of crayfish chitosan, but chemical degradation during film processing limited this effect in the film matrices. Therefore, crayfish-derived biopolymers and films are promising for incorporation into functional foods and active packaging and support shell waste reduction, offering a viable strategy for managing and valorizing invasive species.

## 1. Introduction

Nowadays, from a global perspective on environmental protection, there are new issues to address: the continuous increase in plastic waste, the global waste generated by the food and green sectors, and the increasing trend in non-native species invasion. The continuous increase in plastic waste has led to the development of biodegradable/compostable materials from renewable resources. In line with the European Bioplastic Organization on Packaging, bioplastics account for approximately 1% of worldwide annual plastic production [1], and packaging accounts for almost 65% of the total bioplastics market. Among the natural molecules used to form films for packaging applications, chitin is the second-most abundant polysaccharide after cellulose. Chitin is the precursor of chitosan, and it is mainly isolated from crustacean shells. Chitosan is a biodegradable, biocompatible, chemically stable, antimicrobial and renewable polymer [2]. Therefore, it has been extensively used as a film-forming material across various sectors [3], including food packaging [4]. Even though chitosan has inherent antioxidant and antimicrobial activities, these may not always be sufficient to prevent intense microbial growth and oxidation in the environment. Therefore, the occurrence of antioxidant and antimicrobial compounds improves the quality of biomaterials [5].

Crayfish shells are an excellent source of chitin [6]. Freshwater crayfish are a highly diverse group of decapod crustaceans with over 640 species, some of which are invasive and harmful to fragile freshwater habitats [7]. Invasive species, intentionally or unintentionally introduced outside their native habitat by humans or human-mediated activities, are considered one of the main threats to biodiversity globally [8]. They disrupt freshwater ecosystem functions, inducing food web changes and reductions in water quality and macrophyte biomass, and also potential pathogenicity [9].

Astaxanthin is the primary and most valuable carotenoid in crayfish shells [10], and is of great scientific and commercial interest, as it is an active health-promoting molecule of natural origin with strong prospects for application [11]. It has been mostly applied in the cosmetic and food industries [12,13]. Various methods, such as enzymatic, fermentation, and chemical extraction processes, have been proposed for obtaining astaxanthin from crayfish shells [14,15].

So far, few studies have been conducted on the production of astaxanthin-added materials using non-commercial, crude astaxanthin derived directly from crayfish shell waste [16].

The use of crustacean waste aligns with the principles of sustainable consumption and production and the advancement of tools and solutions in line with the circular economy. By repurposing a waste product that would otherwise have been incinerated, we not only give it value but also foster a culture centered on its resourceful management. Converting waste products into high-value consumer goods will enhance resource efficiency and promote a culture of reuse and valorization. Notably, this study marks the first use of the shells of the invasive spiny-cheek crayfish *Faxonius limosus* to create bioactive films that are promising for food packaging. Hence, we hypothesized that *Faxonius limosus*-based products, i.e., chitin and chitosan biopolymers and bioactive, compostable films, have better physicochemical, mechanical, and biological properties than commercial products, thereby enhancing their market appeal. We also hypothesized that *F. limosus*-based products, naturally containing astaxanthin, have better antioxidant properties than the astaxanthin-deprived ones. The results gathered in this study may also provide a new perspective on managing other biowastes. Therefore, the overall objectives were (a) to obtain and characterize chitin, chitosan, and chitosan-based films from *F. limosus*; (b) to compare their characteristics with those of on-market products; (c) to develop a process to keep astaxanthin preserving its antioxidant properties; and lastly, (d) to propose an innovative approach to manage and to valorize the invasive crayfish *F. limosus* in Central and South America, where invasive species are native and there are millions of them, with millions of carcasses to dispose of. The latter could be exploited as a new, economically sustainable, environmentally friendly alternative to the incineration and disposal of organic waste [17].

## 2. Results and Discussion

### 2.1. Fourier-Transform Infrared Spectroscopy (FTIR)

FTIR is a powerful tool for studying the physicochemical properties of chitosan. Attenuated Total Reflectance (ATR) mode was performed on commercial chitosan, chitin and chitosan from crayfish, and chitosan-based films. All the samples were stored at room temperature in a desiccator with silica gel before testing.

**Figure 1:**
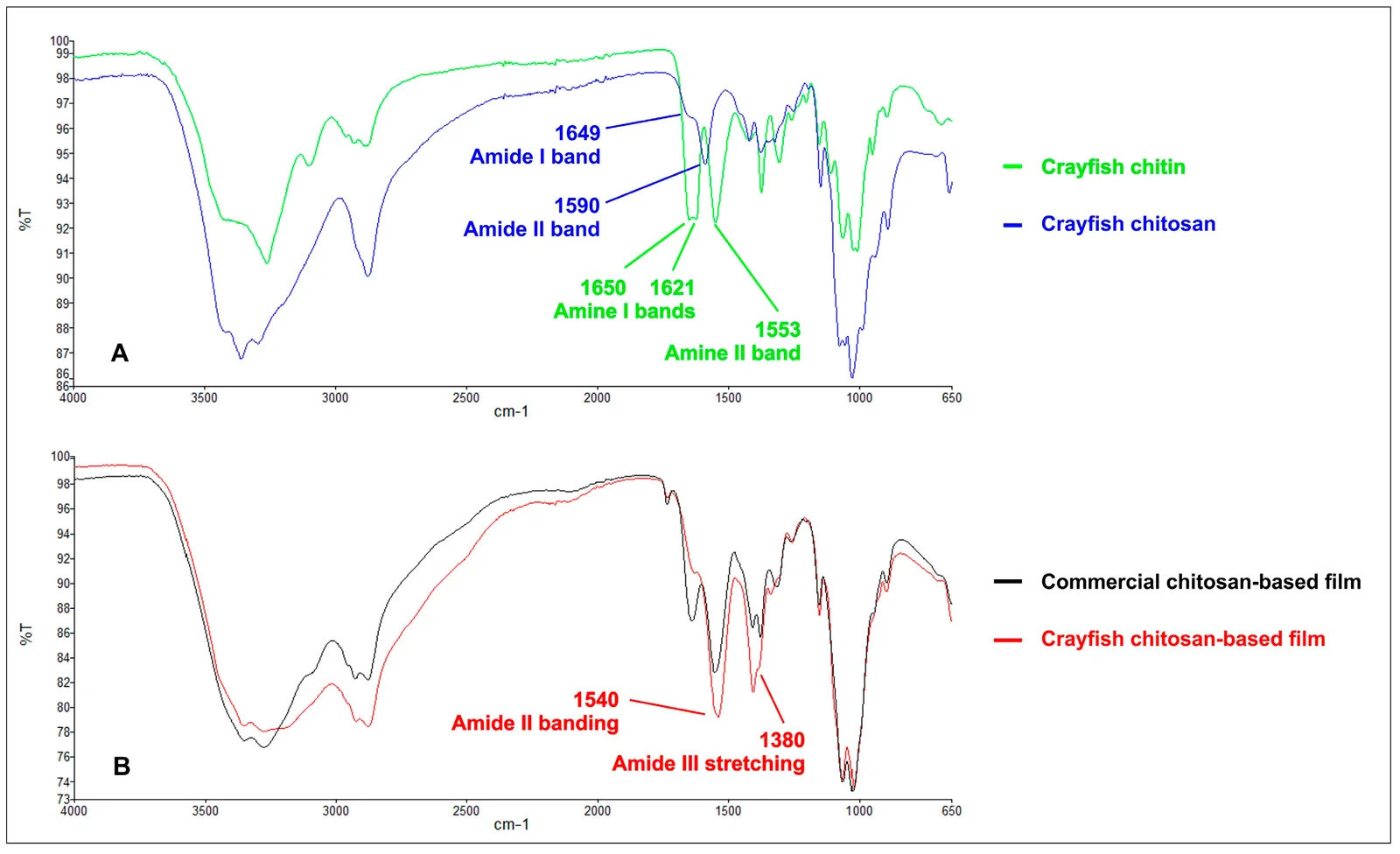
FTIR-ATR of commercial and crayfish products. (**A**) Crayfish chitin and Crayfish chitosan. (**B**) Commercial chitosan-based films and Crayfish chitosan-based film.

The FTIR spectra of chitin and chitosan from crayfish, commercial and crayfish chitosan-based films are shown in Figure 1.

In the FTIR spectrum of crayfish chitosan, two characteristic bands were evident. The presence of residual N-acetyl groups was confirmed by the bands at 1649 cm^−1^ associated with the stretching of ν(C=O) in the NHCOCH3 group (Amide I band), and the band at 1590 cm^−1^ associated with the bending of ν(NH2) in the NHCOCH3 group (Amide II band) [18]. In the FTIR spectrum of crayfish chitin, the Amide I band was observed with a wavenumber between 1600 and 1500 cm^−1^ associated with intermolecular hydrogen bonds [19]. The band at 1375 cm^−1^ corresponds to the stretching deformation of the CH_3_ group. Two peaks at 1620 and 1650 cm^−1^ assured the formation of chitin in the α-form [20,21].

During the deacetylation process (conversion of chitin to chitosan), the removal of acetyl groups causes the Amide II band of chitin (observed around 1550 cm^−1^) to decrease, nearly disappearing, while giving rise to a new primary amine scissoring band at approximately 1590 cm^−1^ in chitosan.

In the FTIR spectra of chitosan-based films, the broad band between 3000 and 3600 cm^−1^ corresponded to the stretching vibration of O-H and N-H groups. The bending of N-H (amide II) was assigned to the 1540 cm^−1^ peak and the stretching of C-N (amide III) to the 1380 cm^−1^ peak [18,22].

### 2.2. Differential Scanning Calorimetry (DSC)

**Figure 2:**
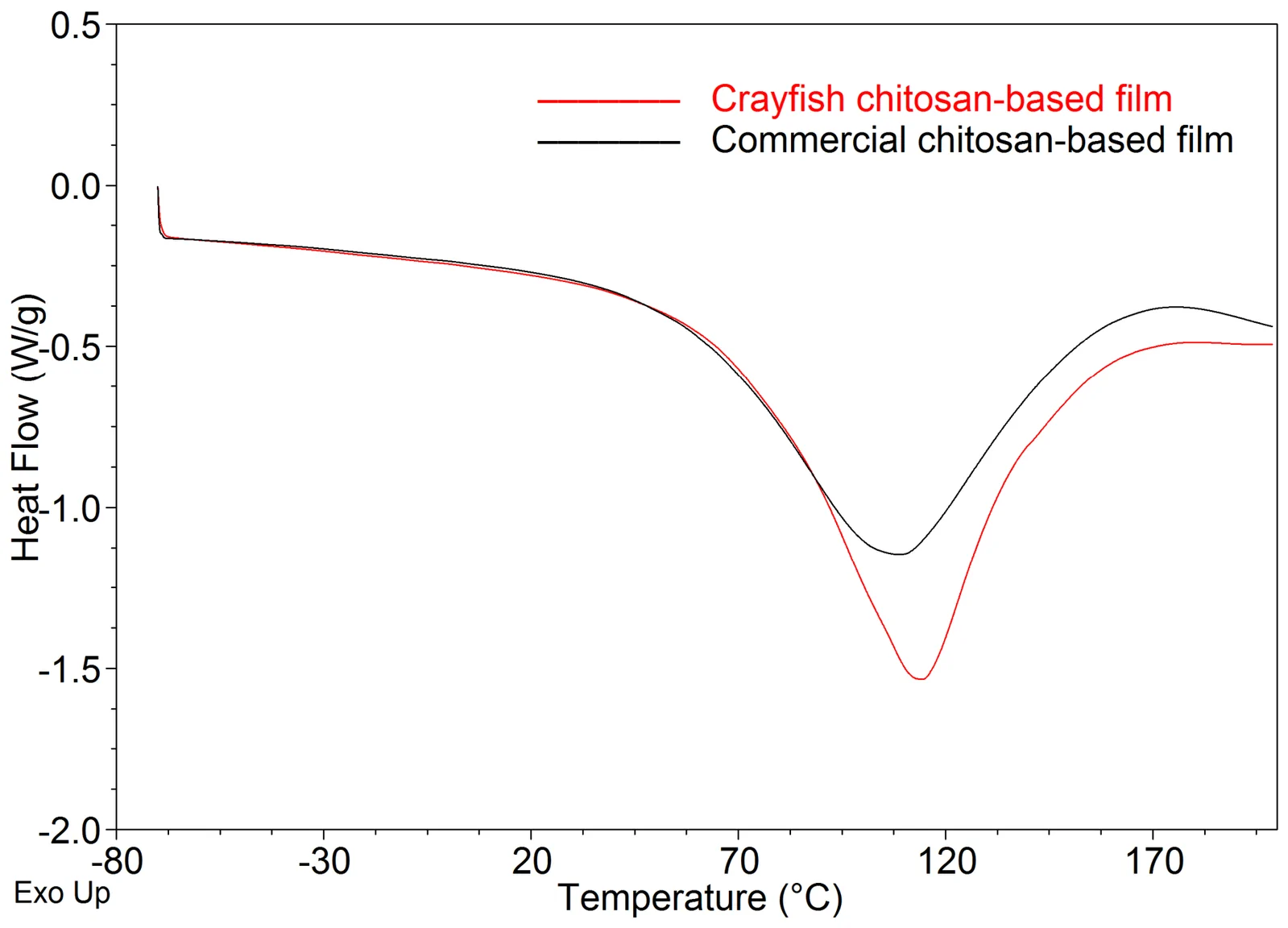
DSC thermograms of commercial chitosan-based film and crayfish chitosan-based film.

DSC scans (Figure 2) and data for the commercial and crayfish chitosan-based films are reported.

The two samples displayed a similar thermal behavior with the exhibition of endothermic peaks. For the commercial chitosan-based film, a T_m_ of 108.3 °C and ΔH_m_ of 305.6 J/g were found. The observed T_m_ value is in agreement with the T_m_ of chitosan films reported in the literature [23]. For the crayfish chitosan-based film, a T_m_ of 113.6 °C and a ΔH_m_ of 344.3 J/g were found. In recent studies, Quiao et al. demonstrate that the endothermic peak at about 105 °C in the DSC thermogram of acetic acid film can be assigned to a cooperative water mediated melting of chitosan. Furthermore, this transition also depends on the pan type of capsule used; in particular, hermetically sealed capsules ensure that the endothermic contribution can be attributed exclusively to the melting of the chitosan. The results highlighted that crayfish chitosan-based films could a have higher molecular weight and/or a more crystalline structure compared to commercial ones. Further, glass transition temperatures (T_g_) were clearly distinguished in Dynamic Mechanical Analysis (DMA) analyses.

### 2.3. Thermo Gravimetric Analysis (TGA)

Chitin and chitosan obtained from crayfish, commercial chitosan, and chitosan-based films were stored at room temperature before testing. The weight loss (% by weight) was determined from the TGA heating scan.

**Figure 3:**
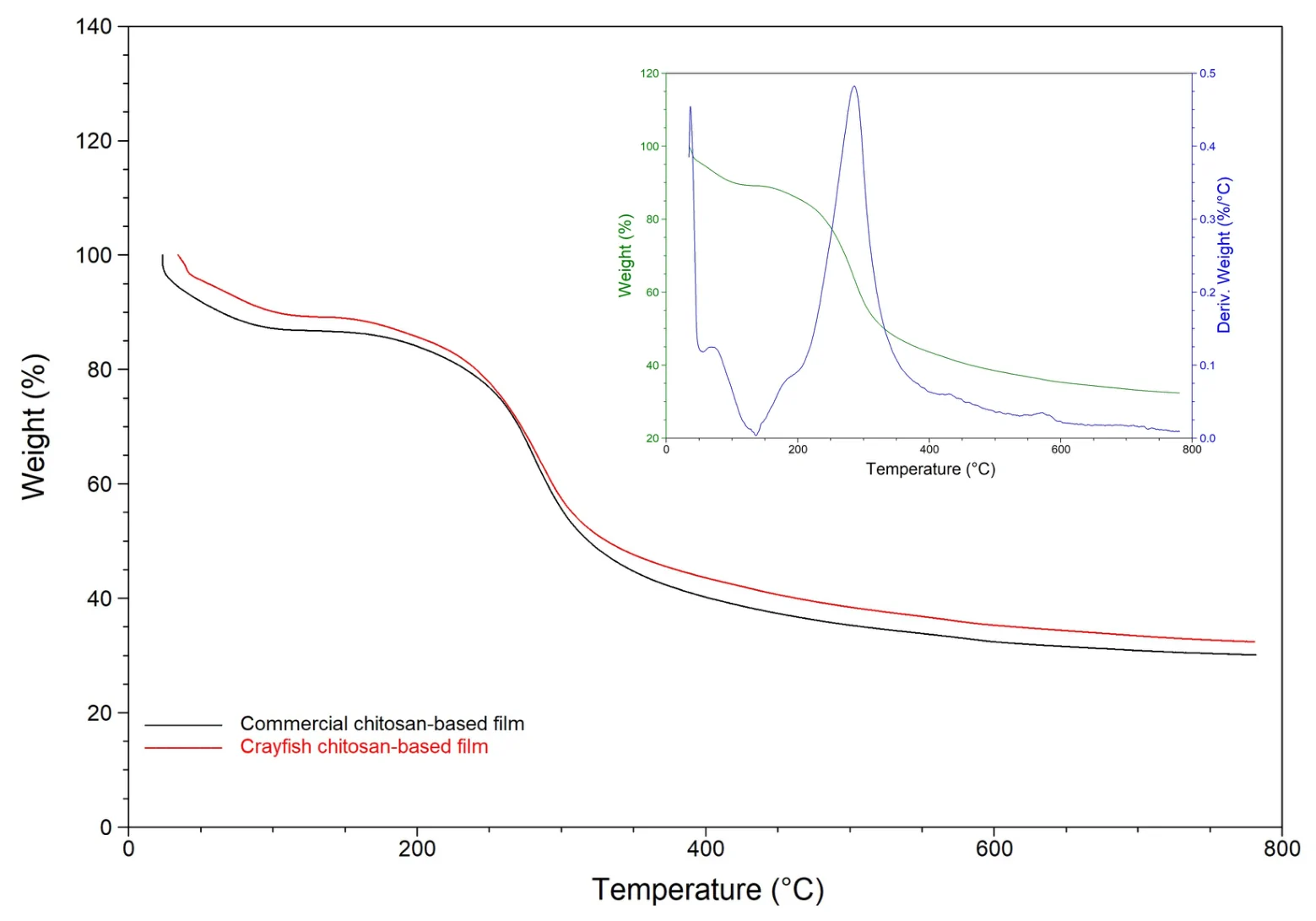
TGA curves of commercial and crayfish chitosan-based film. The inset shows the TGA-derived curve for the crayfish chitosan-based film.

**Figure 4:**
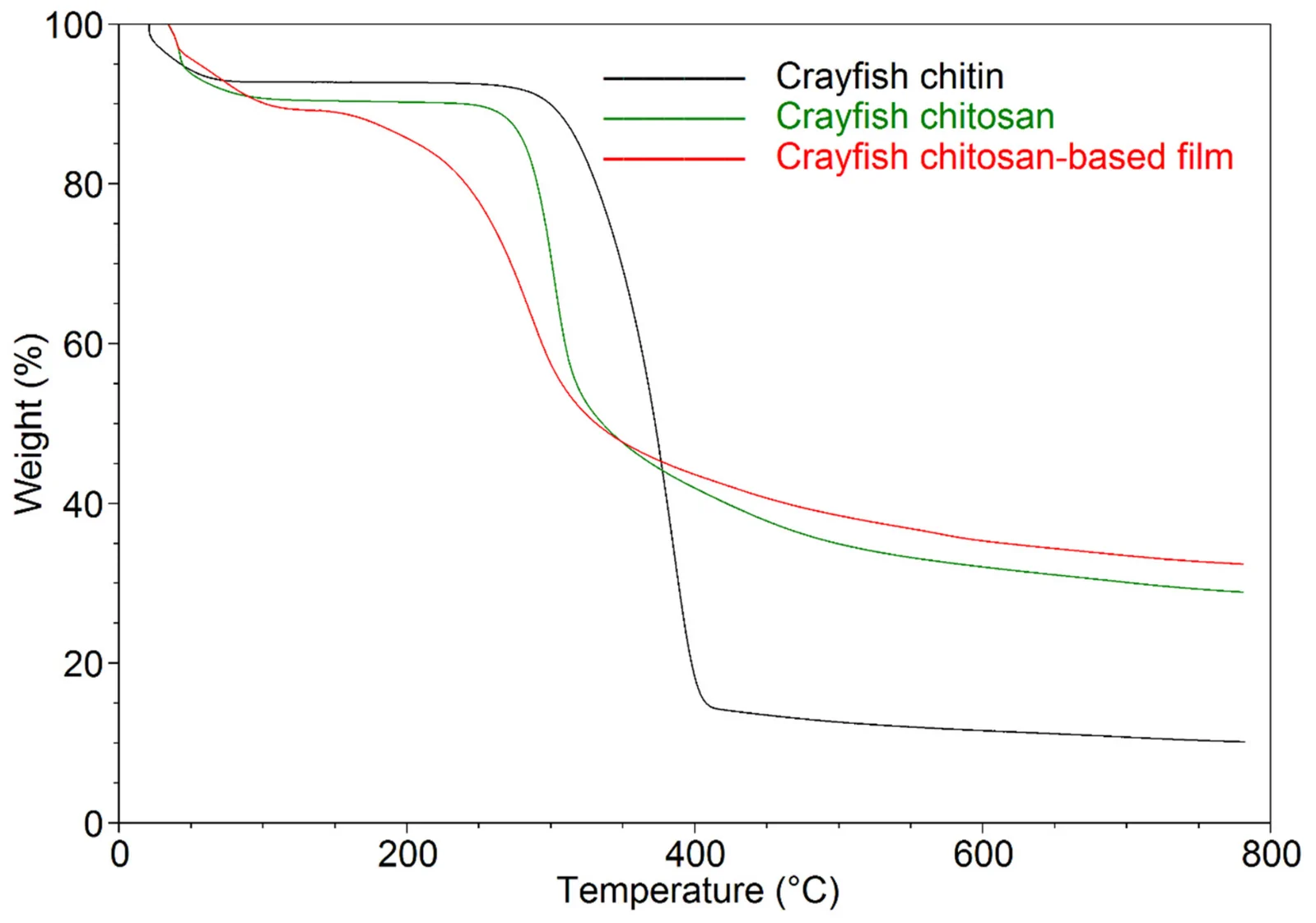
TGA curves of chitin, chitosan, and chitosan-based films from crayfish.

The TGA data (Table 1) and curves (Figure 3 and Figure 4) of the samples are also reported.

A similar thermal degradation behavior was observed for all crayfish samples and chitosan-based films. The thermogravimetric profiles showed two degradation steps. Firstly, a slight weight loss was recorded between 30 °C and 125 °C, while an abrupt change in weight loss was observed at higher temperatures. The weight loss up to 125 °C can be attributed to moisture, while the second is due to material degradation. The weight loss pattern of the two types of films during heating revealed that the chitosan-based films lost most of their weight between 140 and 370 °C (Figure 3). This result could be associated with a complex decomposition process of acetylated and deacetylated units of chitosan [22]. Further, the commercial chitosan-based film shows lower thermal stability up to 262 °C and is more degraded than the crayfish chitosan-based film at 800 °C.

Comparing crayfish products (Figure 4), chitin exhibited sharp degradation between 250 and 410 °C, chitosan between 240 and 450 °C, and the film between 130 and 400 °C. So, the chitin demonstrated a higher thermal stability than the corresponding chitosan, as reported by Abdou [24].

Moreover, data reported in Table 1 highlighted that the initial degradation temperatures (T_i_) and the temperature at the maximum rate of weight loss (T_p_) are in the order of chitin > crayfish chitosan > commercial chitosan > crayfish chitosan-based film > commercial chitosan-based film.

### 2.4. Dynamic Mechanical Analysis (DMA)

**Figure 5:**
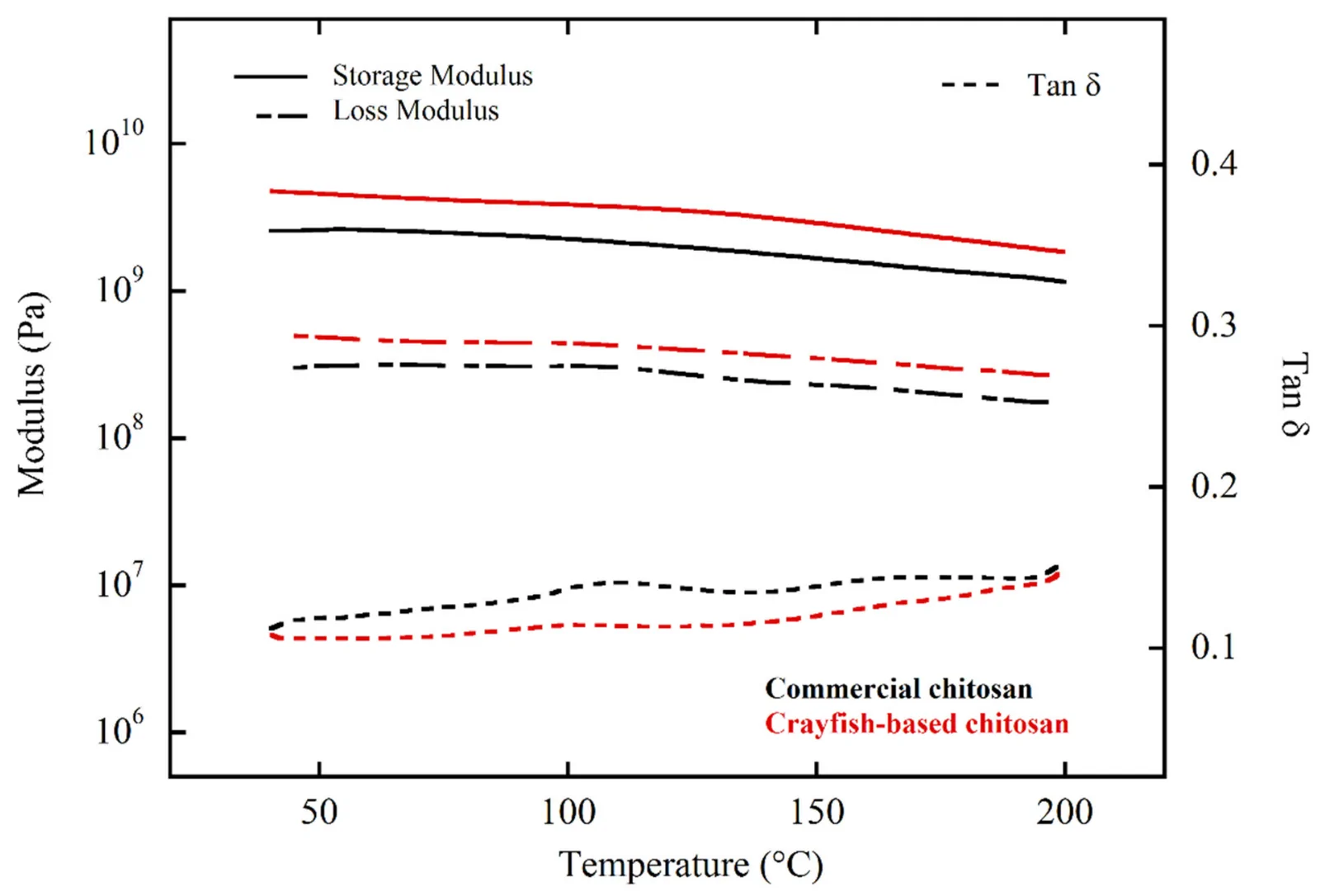
Plots of storage and loss moduli, and Tan δ versus temperature of crayfish-derived chitosan and commercial (reference) chitosan.

Dynamic Mechanical Analysis was selected as the primary method for mechanical comparison because it provides highly sensitive and quantitative information on stiffness, viscoelastic behavior, molecular mobility, and thermal transitions. These parameters are directly linked to the structural organization of chitosan-based films and are particularly informative when comparing materials obtained from chitosan with different molecular weights, degrees of acetylation or processing histories. The dynamic mechanical properties of the crayfish chitosan-based film showed a similar pattern to the commercial chitosan-based film in terms of temperature dependence but exhibited higher values of both loss modulus (0.49 GPa versus 0.29 GPa at 40 °C) and storage modulus (4.6 GPa versus 2.6 GPa at 40 °C), evidencing a higher stiffness (Figure 5). The significantly higher storage and loss moduli observed for the crayfish-derived films indicate a more rigid and mechanically responsive polymer matrix than the commercial reference.

Tan delta curves were also very similar, with slightly more pronounced thermal transitions than those of commercial chitosan. The glass transition temperature (T_g_) of crayfish chitosan-based film was 98.7 °C, while commercial-based chitosan film showed a T_g_ at 109.2 °C. In both materials, the tan delta trend increased with temperature, in accordance with findings reported by other authors [25,26]. The degradation process above 130 °C was manifested by a yellowish coloring after sample extraction from the testing chamber and was also confirmed by the TGA curves.

### 2.5. Water Vapor Permeability (WVP) Tests

**Figure 6:**
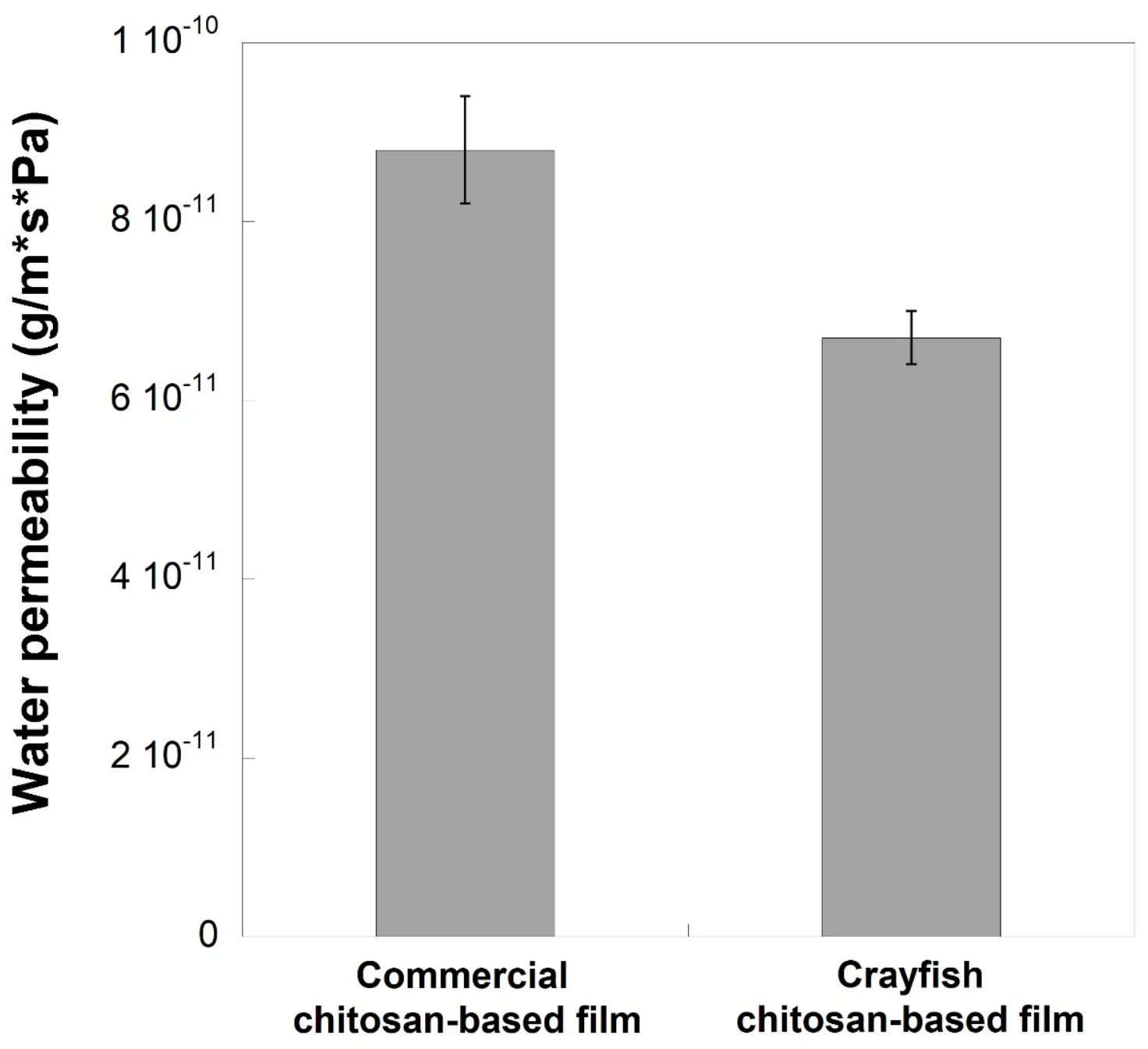
WVP (g/m s Pa) values of commercial and crayfish chitosan-based films.

WVP is the most extensively studied property of films designed for food packaging because of water’s crucial role in food deterioration. The WVP values measured at 25 °C for commercial and crayfish chitosan-based films at an RH gradient of 0–50% are shown in Figure 6. Film thickness was measured at five different locations on each independently cast film, resulting in average values consistent across the three batches prepared and ranging between 0.03 and 0.07 mm. After drying, all films were stored in sealed plastic bags until testing, ensuring uniform pre-conditioning. WVP measurements were performed at 25 °C under a controlled 0–50% RH gradient, a condition commonly used to evaluate moisture transport in hydrophilic biopolymers.

These results are consistent with previously published data for similar materials tested under similar temperature and RH gradient conditions [27]. The WVP values reported in Figure 6 were calculated by the instrumentation software taking into account the actual measured thickness of each tested specimen. Replicate measurements showed low variability among specimens, confirming the reproducibility of the casting procedure and supporting the barrier performance trends observed in Figure 6. It is also evident that the WVP of crayfish chitosan-based film was lower than that of commercial chitosan-based film. Since the film preparation conditions are the same, the different WVP values could be attributed to differences in the molecular weight (Mw) and degree of deacetylation (DD) of the commercial and crayfish chitosans [28]. Some authors reported that the Mw did not influence the WVP [29,30]. However, Liu and co-authors [23] found that an increase in Mw caused decreased WVP values. This behavior was attributed to the low Mw chitosan presenting a lower amount of bond monomers, which facilitates water vapor diffusion through the film. The dependence of the film properties on DD was inverse in relation to the same dependence on Mw. Therefore, crayfish chitosan should have a higher Mw than commercial chitosan, in accordance with the higher melting temperature observed in the DSC test. Crayfish chitosan probably had lower DD than the commercial chitosan used in this study.

### 2.6. Morphological Analysis

**Figure 7:**
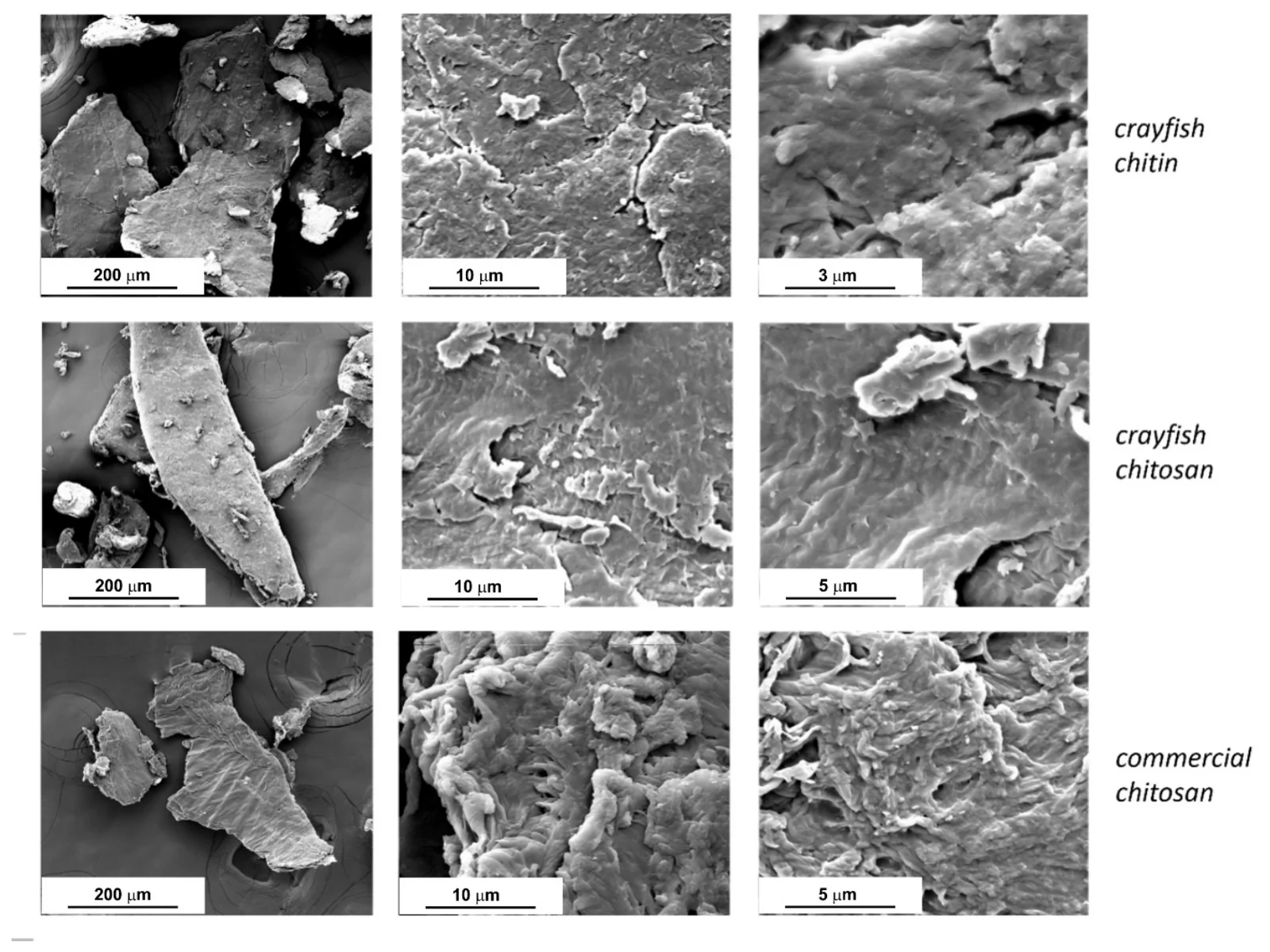
SEM micrographs of chitin and powder from crayfish and commercial chitosan at various magnifications: 500×, 10,000×, 20,000×, and 40,000×.

The surface morphologies of the chitin, chitosan powders and films from crayfish and commercial chitosan were examined through Scanning Electron Microscopy (SEM) analysis. SEM images with various magnifications (500×, 10,000×, 20,000×, and 40,000×) are presented in Figure 7.

**Figure 8:**
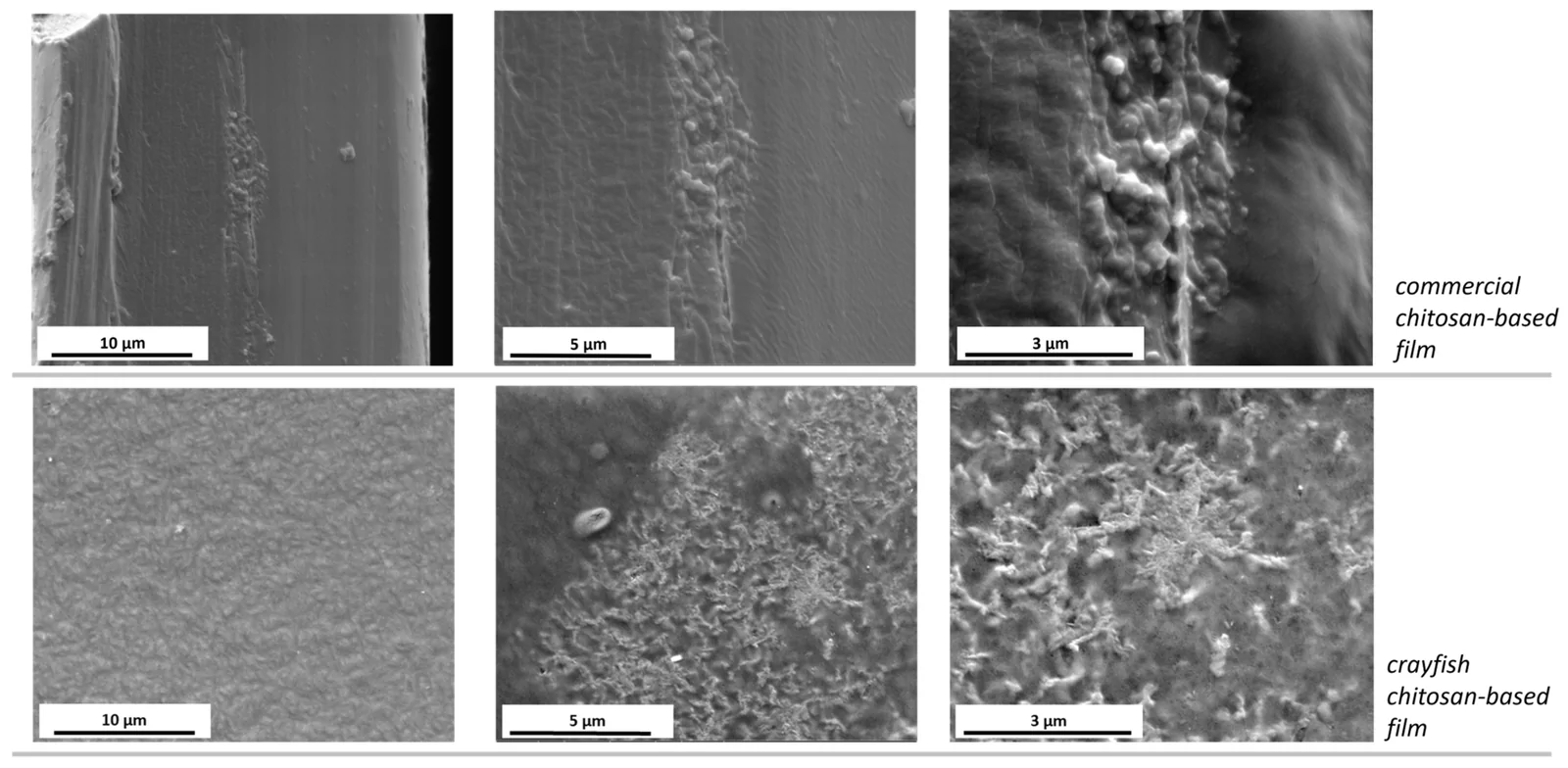
SEM micrographs of commercial and crayfish chitosan-based films at various magnifications: 10,000×, 20,000×, and 40,000×.

SEM analyses were performed on multiple independent samples, and the observed morphological features were consistent across all replicates, confirming the reproducibility of the results. In general, chitin and chitosan may be classified into three surface morphologies: (1) with porosity and microfibrillar structure, (2) without porosity or microfibrillar structure, and (3) with only a microfibrillar structure [31]. The results of this study indicated that there were no nanofibers or nanopores on the surfaces of crayfish-based chitin and chitosan. In a previous study, it was reported that chitosan from the shell of an endemic Chilean crayfish (*Parastacus pugnax*) has a smooth surface [32]. On the contrary, Kucukgulmez [33] obtained crayfish-based chitin, which has a uniform nanofiber structure from *Astacus leptodactylus* shell waste. Kadak [34] revealed that the surface morphology of crayfish-based chitosan from *A. leptodactylus* consisted of filamentous structures whose number decreases as the levels of chitosan deacetylation increase. The surface morphologies of the chitin and chitosan of this crayfish species exhibited smooth and film structures different from the fibrillar and porous chitin and chitosan in other crustaceans (see commercial chitosan) and insects [35,36]. Therefore, the surface morphology of chitosan varies according to the source species used. In agreement with the literature reports on chitosan films [37], the uniform and smooth surface morphology observed in this study suggests that β-1,3-glucan components may not have been fully removed from the chitin matrix during chemical processing. Figure 8 comprised the SEM images of commercial and crayfish chitosan-based films. Images reveal that commercial chitosan-based film is smooth but with large micron-sized aggregates.

On the contrary, the crayfish chitosan-based film is rough but has fewer aggregates. Moreover, the morphology of both films is without pores, just the opposite of the uniformly distributed pores on the surface of the chitosan film reported by Pereda [38]. However, this phenomenon generally depends on the nature of the chitosan used (degree of acetylation and molecular weight) and on the evaporation rate of the solvent during film formation [39]. The films produced in this study have a pore-free morphology, resulting in improved mechanical and barrier properties compared to chitosan films found in the literature. This makes them better promising candidates for food packaging. Although SEM provides qualitative information, it offers essential morphological evidence that complements the structural and functional characterization of the materials, supporting the interpretation of their mechanical and barrier properties.

### 2.7. Spectrophotometry and Chromatographic Analysis of the Crayfish Shell

**Figure 9:**
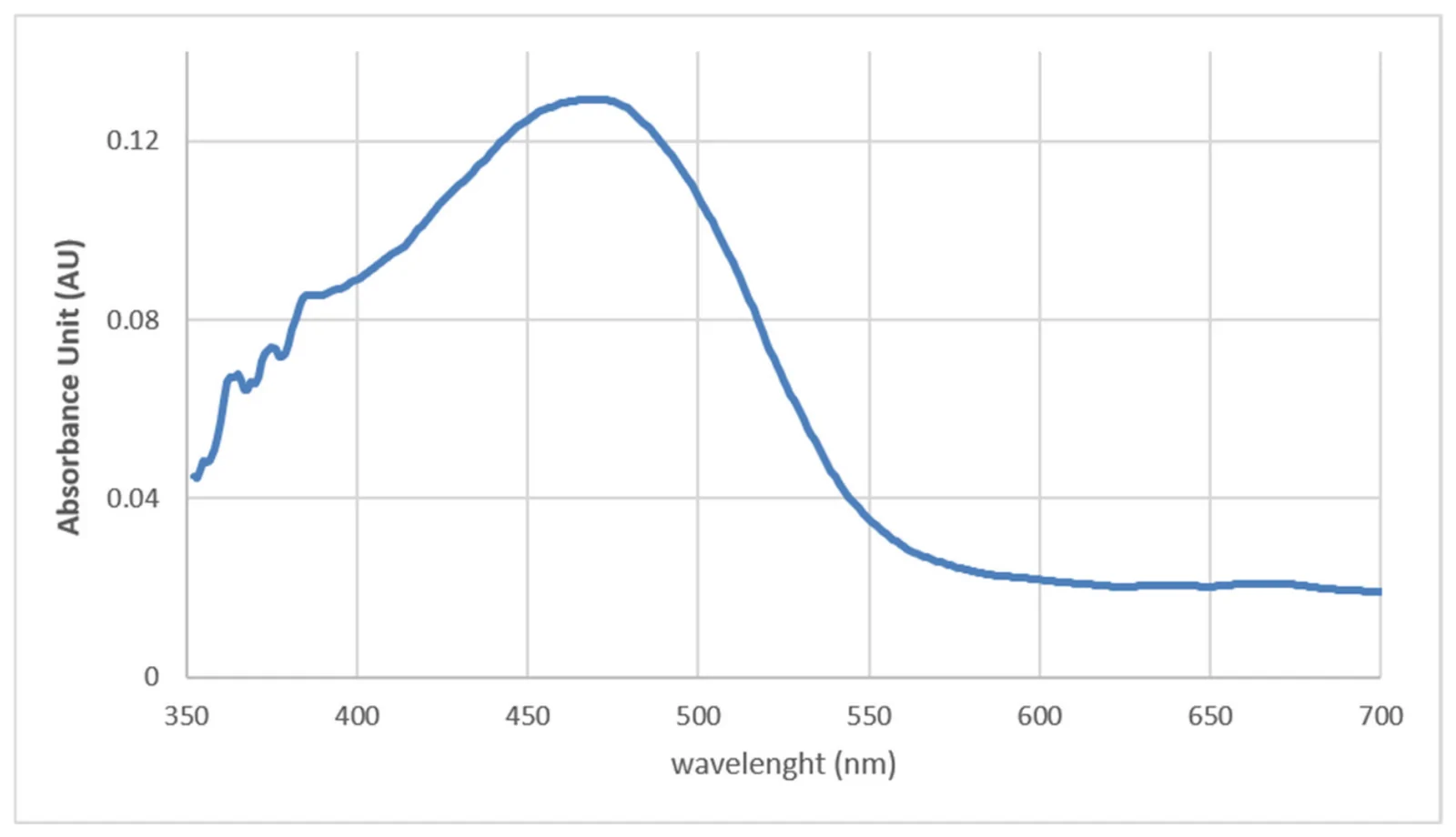
Absorption spectra of the acetone extract (Acetone: Water 90:10) of the crayfish shell.

A spectrophotometric analysis has been performed to characterize the pigments extracted in acetone: crayfish shells used to produce the chitosan (see Step 3: Chitin partial discoloration—Astaxanthin removal). The UV spectra (Figure 9) were obtained from an acetone extract (Acetone: Water 90:10) that matches the one obtained for astaxanthin carotenoid.

**Figure 10:**
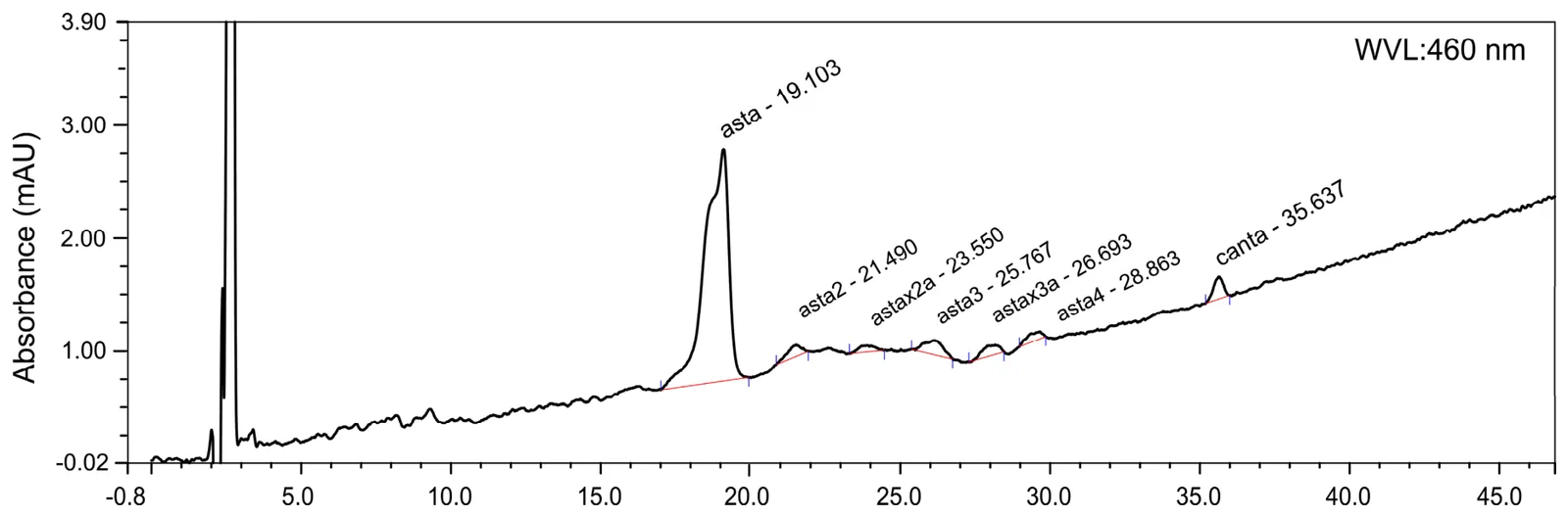
HPLC chromatogram of an acetone: water (90:10) extract from crayfish shell.

The same extract was also used for injections in an HPLC chromatographic system to check for the possible presence of isomers or other carotenoids in the crayfish shell. In Figure 10, the chromatogram obtained from the same acetone: water extract is shown, confirming that astaxanthin is the major carotenoid present, along with a few other isomers. Peak identification was based on retention time and spectral comparison with a reference pigment from a zooplankton extract.

UV and HPLC results confirm that the discoloration step removes astaxanthin from the biopolymer.

To assess whether astaxanthin undergoes degradation during the preparation of the chitosan film, chitosan derived from non-discolored chitin was dissolved in the same acetic acid solution employed for film fabrication. The resulting solutions were analyzed after three different dissolution periods: 1, 3 and 24 h.

**Figure 11:**
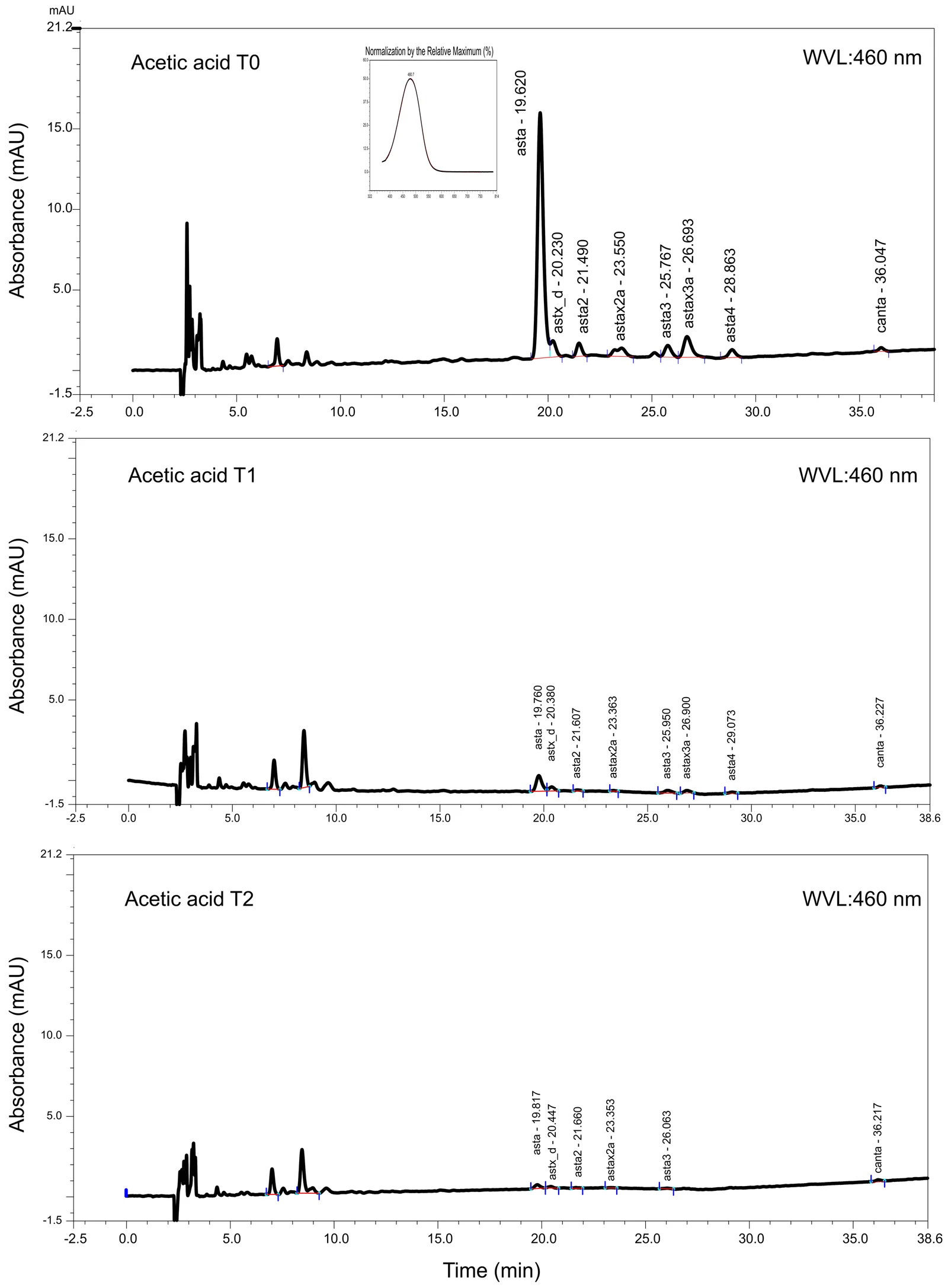
Effect of acetic acid treatment on astaxanthin content in crayfish shells at three different time intervals.

Figure 11 shows the changes in pigment composition induced by acetic acid treatment at three different time intervals for the astaxanthin present in the crayfish shell. The results indicate substantial astaxanthin degradation; however, even after longer exposure times, a small amount of carotenoid remains detectable.

### 2.8. Radical Scavenging Activity

**Figure 12:**
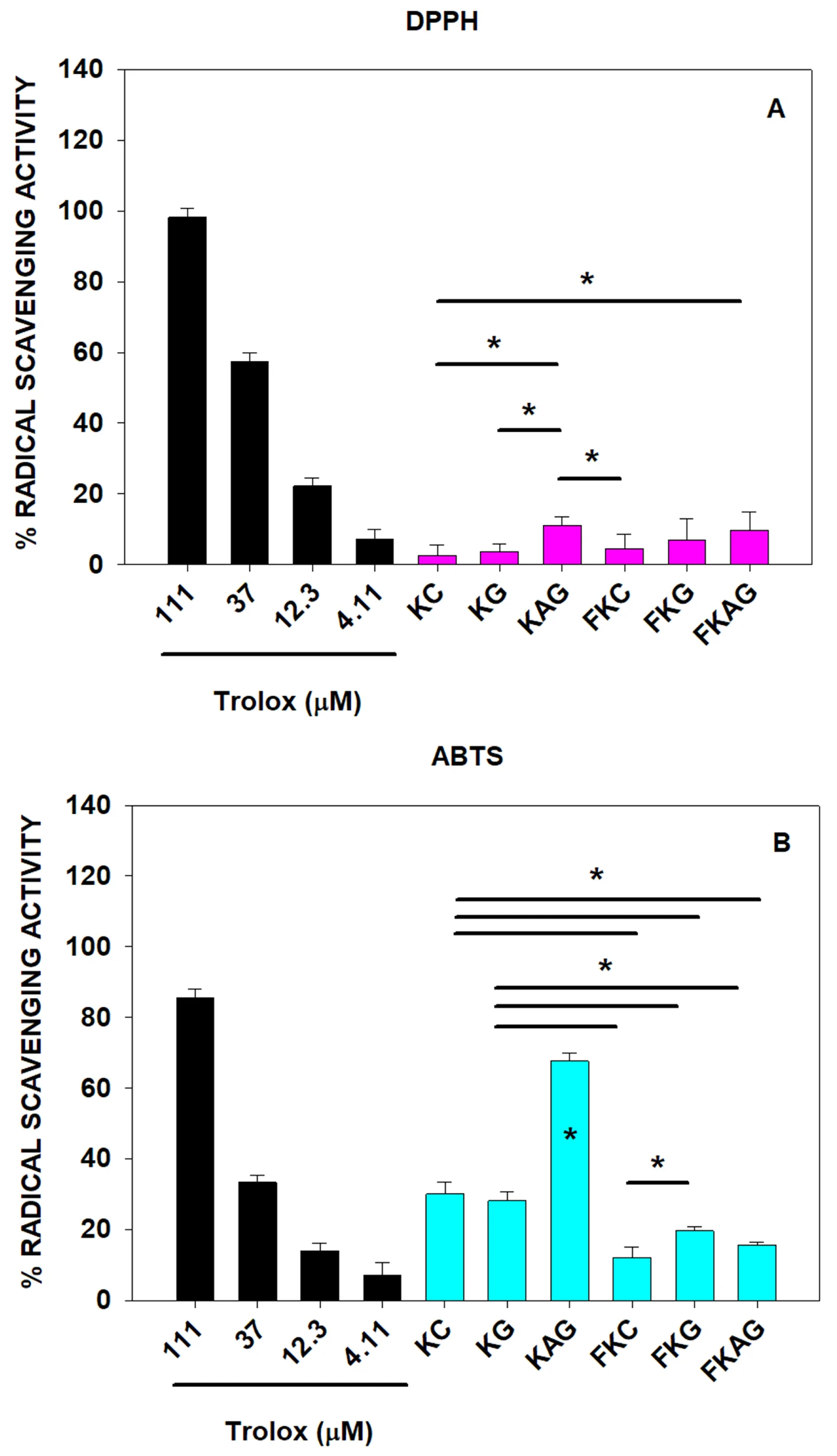
Antioxidant properties of commercial and crayfish-derived materials. Vertical bars represent the percentage of radical scavenging activity for each chitosan sample as determined by the DPPH (**A**) and ABTS (**B**) assays. Error bars indicate the standard deviation. * Significant difference (*p* < 0.05). KC, Commercial chitosan; KG, Crayfish chitosan; KAG, Crayfish chitosan + astaxanthin; FKC, Commercial chitosan-based film; FKG, Crayfish chitosan-based film; FKAG, Crayfish chitosan + astaxanthin film.

Two radical scavenging assays, 2,2-diphenyl-1-picrylhydrazyl (DPPH) and 2,2′-az-inobis (3-ethylbenzothiazoline-6-sulfonic acid) (ABTS), were performed to characterize the antioxidant properties of the chitosan powders (with and without astaxanthin) and film samples and define the potential contribution of astaxanthin. The two tests showed different reactivity to the samples. The analysis of the two antioxidant test results showed that the sample with crayfish chitosan + astaxanthin had significantly higher antioxidant activity than the commercial chitosan, crayfish chitosan, and commercial chitosan-based film (Figure 12A,B, Table 2). The ABTS assay also showed significantly increased antioxidant activity in the commercial and crayfish chitosan samples compared with all film samples (Figure 12B, Table 2). The relative responses of commercial chitosan versus the crayfish chitosan–astaxanthin film, and of the commercial chitosan-based film versus the crayfish chitosan-based film, differed between the DPPH and ABTS assays (Figure 12A,B). Compared with crayfish chitosan without astaxanthin, crayfish chitosan containing astaxanthin showed increases in mean radical scavenging activity of 140% in the ABTS assay and 93.8% in the DPPH assay.

## 3. Materials and Methods

### 3.1. Reagents

NaOH, HCl, and other analytical-grade reagents were purchased from Sigma-Aldrich, Milano, Italy, as well as high-molecular-weight chitosan (Deacetylated chitin, Poly (D-glucosamine)) used as a reference. 2,2-diphenyl-1-picrylhydrazyl (DPPH, cat. num. D9132), 2,2′-azinobis(3-ethylbenzothiazoline-6-sulfonic acid) diammonium salt (ABTS, cat. num. A1888), potassium peroxydisulfate (cat. num. 216224) and 6-Hydroxy-2,5,7,8-tetramethylchroman-2-carboxylic acid (Trolox, cat. num. 238813) were purchased from Merck KgaA (Darmstadt, Germany). Acetic acid, spectrophotometry and HPLC reagents: methanol and acetone (HPLC grade) were purchased from VWR, Milano, Italy. Milli-Q water (ELGA LabWater, Veolia, Lane End, UK) was used for solvent preparation for chromatography and for extraction.

### 3.2. Crayfish Collection

Crayfish (*Faxonius limosus*) waste derived from specimens collected in 2022 during routine monitoring and sampling of the littoral zone of three Italian lakes (Maggiore, Mergozzo, and Orta, NW Italy) [40] was used as raw material for chitosan production. After trapping, the specimens were transported to the laboratory and frozen at −20 °C within 6 h of sampling.

### 3.3. Chitosan Production from Faxonius limosus Shell

The conventional methods for extracting chitin and producing chitosan involve a decolorization step that results in the loss of astaxanthin, an important antioxidant. To harness the antioxidant properties of astaxanthin and preserve astaxanthin in chitin and chitosan, we removed the decolorization step that uses acetone and minimized the temperatures and reaction times to prevent astaxanthin degradation. As a result, the chitin and chitosan retained the natural antioxidant found in the carapace. Additionally, we employed gentler chemical processes and eliminated the use of organic solvents for astaxanthin removal, thus making the entire process more sustainable.

Shell preparation

After defrosting, the shell was separated from the animal tissues and thoroughly cleaned under running water by scrubbing it with a brush. Then, to remove all the traces of tissue, the shells were heated in water (70 °C) for 15/20 min, reduced into small pieces and washed repeatedly with running water to eliminate further residues of animal tissues. Finally, the material was dried for 1 h at 80 °C and coarsely ground with a blender.

Chitin extraction and chitosan preparation were carried out in three or four steps (for astaxanthin removal), following the procedure of El-Naggar et al. [6], modified as described below.

Step 1: Chitin extraction—Demineralization

The coarse shell powder was suspended in 1 M HCl with a solid-to-solvent ratio of 1:15 (*w*/*v*) at room temperature and kept under magnetic stirring until the effervescence due to CO_2_ production disappeared and the pH was still acidic. The sample was washed with distilled water until it had a neutral pH and dried at 45 °C for 18 h.

Step 2: Chitin partial purification—Deproteinization

To minimize the protein content, the sample obtained in step 1 was suspended in 3.5% NaOH solution with a solid-to-solvent ratio of 1:39 (*w*/*v*) and kept at 42 ± 1 °C for 3.5 h, under magnetic stirring. The powder was then washed with distilled water until it had a neutral pH.

Step 3: Chitin partial discoloration—Astaxanthin removal

This step was primarily aimed at removing the astaxanthin pigment. For this purpose, the sample was suspended in acetone under stirring and then separated from the colored astaxanthin solution, which was discarded. The astaxanthin extraction procedure was repeated four times. Finally, the recovered powder was dried at room temperature and finely ground with a laboratory mini grinder (Pulverisette 23, Fritsch GmbH, Idar-Oberstein, Germany).

This step was not applied to chitin samples prepared to produce astaxanthin-containing chitosan and astaxanthin-chitosan films.

Step 4: Chitin deacetylation—Chitosan production

Chitin was suspended in 50% NaOH solution at a solid-to-solvent ratio of 1:10 (*w*/*v*) and heated under magnetic stirring in a boiling water bath at about 100 °C for 6 h. The obtained chitosan suspension was cooled in a water bath at room temperature, washed with distilled water until neutral pH and dried at room temperature in a desiccator with silica gel. The sample was stored in polyethylene bags for further use.

The process yield was calculated in triplicate (*n* = 3) based on the dry weight of the isolated chitosan relative to the starting dry biomass of the carapace, yielding 15 ± 1%.

### 3.4. Chitosan-Based Film Production

#### 3.4.1. Preparation of the Solution

Chitosan was oven-dried to a constant weight. Then 1 g chitosan was dissolved in 100 mL water and 2 mL acetic acid by stirring the initial suspension for 24 h at room temperature. Air bubbles were eliminated by keeping the solution at room temperature for 2 h [41].

#### 3.4.2. Film Casting and Drying

Aliquots of the resulting clear chitosan solution were cast onto polystyrene Petri dishes and allowed to dry at room temperature for 7 days to obtain films. After drying, the films were carefully peeled from the Petri dishes and stored in sealed plastic bags until further use. Three fully independent batches of films were prepared for each formulation.

All films were transparent, homogeneous, and free of visible air bubbles. Film thickness was measured using a digital micrometer (Mitutoyo, Kanagawa, Japan) at five different locations on each film sample cut from the cast slab to assess thickness uniformity and ensure reproducibility. The average measured thickness ranged from 0.03 to 0.07 mm among the different samples. All measurements were performed on samples cut from three independently prepared films.

### 3.5. Characterization of Chitin/Chitosan Powder and Films

#### 3.5.1. FTIR

A PerkinElmer Frontier FT-IR/NIR spectrometer (Waltham, MA, USA) in attenuated total reflection mode (ATR) was used to collect spectra within a wavenumber range of 4000–650 cm^−1^ with a resolution of 4 cm^−1^ and 32 scans. Both powders and films of the materials under investigation were subjected to spectral analysis. The SPECTRUM version 10.5.1 software was employed for spectrum analysis.

#### 3.5.2. Thermal Analysis

Calorimetry tests were performed by differential scanning calorimetry (DSC Q20, TA Instruments, New Castle, DE, USA) and thermo-gravimetric analysis (TGA Q500, TA Instruments) in a nitrogen atmosphere. The acquired data were analyzed using the TA Instruments Universal Analysis 2000 software. For the DSC tests, hermetically sealed capsules were used and the samples were heated at a rate of 10 °C/min over the temperature range of −70 to 200 °C to obtain the melting temperature (T_m_) and heat of fusion (ΔH_m_ in J/g) of the films. In DSC measurements performed in triplicate, the estimated margins of error are ±0.5 °C for temperature values and ±3% for enthalpy.

TGA analyses were performed on the powders and films at a heating rate of 10 °C/min from 25 to 800 °C. Initial degradation temperatures (T_i_) and the temperature at the maximum rate of weight loss (T_p_) were calculated from the TGA-derived curves.

In TGA measurements performed in triplicate, the estimated margins of error were ±1.5 °C for decomposition temperatures (T_i_ and T_p_) and ±2.5%for mass loss percentages.

#### 3.5.3. DMA Characterization

Thermo-dynamic mechanical properties of films were evaluated with a dynamic mechanical analyzer (DMA model TRITEC 2000, from Triton Technology Ltd., London, UK) in tensile mode, in accordance with ASTM D5026 [42]. The analysis aimed to evaluate the main thermal transitions and the magnitude of storage and loss moduli. Samples were cut from cast films and dried in an oven under vacuum at 100 °C for 3 h to eliminate residual solvent and humidity. The sample size was 20 mm × 9 mm, with a thickness ranging between 0.03 and 0.07 mm. DMA tests were performed in tension mode, with a 1 N preload, a dynamic displacement amplitude of 50 μm, and a frequency of 1 Hz. The temperature ranged from 25 °C to 200 °C (or until the occurrence of a signal drop), and the heating rate was 2 °C/min.

#### 3.5.4. Water Vapor Permeability

The WVP was measured using a high-tech analyzer Mocon (Brooklyn Park, MN, USA) Permatran W 3/31, at 25 °C on a sample area of 5 cm^2^ by setting the relative humidity (RH) at the downstream and upstream sides of the film to 0% and 50%, respectively. Each test was carried out in triplicate, and the average values are reported. To compare these results, all WVP results are referenced to a sample thickness of 0.5 mm.

#### 3.5.5. SEM Analysis

Scanning electron microscopy (SEM) FEI Quanta (Hillsboro, OR, USA) 200 FEG was used to analyze the chitin and chitosan surface, along with chitosan-based films. Before scanning, the chitosan, chitin particles and film samples were coated with a thin layer of gold using a sputter coater (EMS550X). The images were captured at different magnifications using an acceleration voltage of 5 kV.

#### 3.5.6. Spectrophotometry and HPLC Analysis

Pigments were extracted from approximately 1 g of dried (at room temperature) and grounded powder with 5 mL of 90% acetone overnight in the dark under nitrogen atmosphere. The samples were clarified with a centrifugation step (3000 rpm for 10 min). The supernatant was used to quantify chlorophyll derivatives (CD) and total carotenoids (TC) with a UV-VIS spectrophotometer (UV mc2, SAFAS) following Lami et al. [43]. Specific carotenoids of different taxonomic affinities were detected by high-performance liquid chromatography using an Ultimate 3000 system (ThermoFisher, Waltham, MA, USA) equipped with a quaternary pump, an autosampler, and a DAD detector. The elution program and the methods used for pigment identification and quantification followed Lami et al. [44].

#### 3.5.7. Radical Scavenging Assays: DPPH and ABTS

**DPPH assay**

The DPPH method was performed according to the protocol developed by Blois et al. [45]. Briefly, a 0.25 mM DPPH stock solution was prepared by dissolving 1.2 mg DPPH in 12 mL of absolute ethanol. A 5 mM Trolox stock solution was prepared by dissolving 1.2 mg Trolox in 0.96 mL of absolute ethanol. Approximately 20 mg of dry sample or film was mixed with 0.5 mL of a 0.2 mM DPPH ethanolic solution. The Trolox stock solution was diluted in absolute ethanol and mixed 1:4 with a 0.25 mM DPPH solution to prepare positive controls (1000–4.1 mM). All samples were incubated for 30 min in the dark at room temperature under 70 rpm 3D shaking (iShak 3D-5 NXT, Neuation Technologies, Gujarat, India). Absorbance was measured in a 96-well plate using a TECAN F-200PRO plate spectrophotometer (Tecan Trading AG, Männedorf, Switzerland) equipped with a 540 nm filter (10 nm bandwidth). The 0.2 mM DPPH ethanolic solution was used as a control, and absolute ethanol served as a blank. The percentage scavenging activity (% SA) was calculated using the following formula: % SA = [(Ac − As)/Ac] × 100, where Ac = absorbance of the control, and As = absorbance of the test sample. All experiments were carried out in triplicate.

**ABTS assay**

The ABTS cation radical solution was prepared as described by Lee et al. [46]. Briefly, 76.82 mg ABTS was dissolved in 20 mL of bidistilled water to obtain a seven mM stock solution. A potassium persulfate solution was added to the ABTS stock solution to achieve a final concentration of 2.45 mM, and the mixture was allowed to stand for 18 h at room temperature until a dark blue-green color developed.

Once ready, the blue-green ABTS• solution was diluted with ethanol to an absorbance of approximately 0.70 at 750 nm ± 10 nm. Subsequently, 20 mg of the dry sample or film was mixed with 0.5 mL of diluted ABTS• solution. As was done for the DPPH assay, trolox was used to prepare 1000–4.1 mM reference solutions. All samples were incubated for 15 min in the dark at room temperature under 70 rpm 3D shaking (iShak 3D-5 NXT, Neuation Technologies, Gandhinagar, Gujarat, India). Absorbance was measured in a 96-well plate using a TECAN F-200PRO plate spectrophotometer (Tecan Trading AG, Switzerland) equipped with a 750 nm filter (10 nm bandwidth). The diluted ABTS solution was used as a control, and absolute ethanol as a blank. The percentage scavenging activity (% SA) was calculated using the following formula: % SA = [(Ac − As)/Ac] × 100, where Ac = absorbance of the control, and As = absorbance of the test sample. All experiments were carried out in triplicate.

**Trolox-equivalent antioxidant capacity**

The Trolox-equivalent antioxidant capacity was determined by making an inverse prediction from the linear regression between the radical scavenging response and Trolox concentration. The calculated Trolox-equivalent concentration was normalized to the mass of the powder using the formula: TE (µmol/g) = CTE (µmol/L) × V (L)/m (g). In this equation, V represents the total reaction volume of 0.000500 L, and m refers to the powder mass of 0.020 g [47].

The lowest concentration of the Trolox calibrator was 4.115 µmol/L, which corresponds to 0.103 µmol TE/g under the experimental conditions. Any sample responses that fell below the response of the lowest calibrator were reported as <0.103 µmol TE/g.

#### 3.5.8. Data Analysis and Statistics

Data analysis and plotting were carried out using Microsoft Excel 365 (Microsoft Corp., Redmond, WA, USA). To assess the statistical significance of differences between groups, a one-way analysis of variance (ANOVA) followed by Holm–Sidak multiple com-parison tests was applied. Statistical analysis was performed using SigmaPlot 11 software (Inpixon, Palo Alto, CA, USA). The statistical significance was set at *p* < 0.05.

## 4. Conclusions

This study demonstrates a feasible valorization route for shell waste from the invasive crayfish *Faxonius limosus*, converting biomass otherwise destined for disposal or incineration into chitin, chitosan, and chitosan-based films with potential application in active food packaging. By simplifying the conventional chitin/chitosan production protocol, lowering selected processing temperatures, and omitting the discoloration step, the proposed method reduced solvent use and energy demand while enabling partial retention of the naturally occurring carotenoid fraction, mainly astaxanthin.

The crayfish-derived chitosan and films showed promising physicochemical and functional properties. In particular, the crayfish chitosan-based films exhibited higher thermal and mechanical stability, lower water vapor permeability, and a smoother surface compared with commercial chitosan-based reference films. These findings suggest that the chitosan film derived from *F. limosus* shells shows promising potential for the development of biodegradable packaging materials. Astaxanthin played an important role in the antioxidant performance of the recovered biopolymers. Its presence markedly increased the radical scavenging activity of crayfish chitosan powder, confirming the added value of preserving this bioactive compound during shell valorization. However, this antioxidant enhancement was not sustained in the final film matrices. Although the degradation of astaxanthin during the acetic acid dissolution and film-forming steps is a plausible explanation, additional studies quantifying residual astaxanthin and evaluating its release from the film matrix are required to confirm the mechanisms involved.

Overall, the results presented in this work serve as a proof of concept for the integrated exploitation of invasive crayfish shell waste as a source of bioactive biopolymers and biodegradable films. These preliminary findings demonstrate that the developed films represent promising candidates for future application in active food packaging. Further work should focus on optimizing processing conditions to limit astaxanthin degradation, assessing antimicrobial, barrier properties and food preservation performance in real packaging systems, and evaluating the environmental and economic sustainability of the proposed process on a larger scale.

## Figures and Tables

**Table 1:** Thermogravimetric data (in %wt) of commercial and crayfish-based materials.

Materials	Degradation 30–125 °C (%wt)	Degradation 30–800 °C (%wt)	T_i_ (°C)	T_p_ (°C)
Commercial Chitosan	9	69	230	293
Crayfish Chitosan	9	71	241	301
Crayfish Chitin	7	89	258	386
Commercial chitosan-based film	10	69	132	280
Crayfish chitosan-based film	10	68	137	287

**Table 2:** Trolox-equivalent antioxidant capacity of chitosan samples, expressed as µmol Trolox equivalents per gram of sample (µmol TE/g). The lowest Trolox calibrator was 4.115 µmol/L, corresponding to 0.103 µmol TE/g under the experimental conditions. Sample responses below the response of the lowest calibrator were reported as <0.103 µmol TE/g.

Sample Type	Sample Code	Integrated % Scavenging Activity
Commercial chitosan	KC	32.6 ± 4.4
Crayfish chitosan	KG	33.9 ± 4.2
Crayfish chitosan + astaxanthin	KAG	78.8 ± 3.3
Commercial chitosan-based film	FKC	21.1 ± 8.1
Crayfish chitosan-based film	FKG	26.6 ± 6.3
Crayfish chitosan + astaxanthin film	FKAG	25.4 ± 5.1

## Data Availability

The original contributions presented in this study are included in the article. Further inquiries can be directed to the corresponding author.
